# Supplementary material for: LMM24 Encodes Receptor-Like Cytoplasmic Kinase 109, Which Regulates Cell Death and Defense Responses in Rice
Source: Int J Mol Sci. 2019 Jul 2;20(13):3243. doi: 10.3390/ijms20133243 (PMC6651581; doi:10.3390/ijms20133243)
Supplement: Supplementary file 1 [file ijms-20-03243-s001.pdf]

International Journal of *Molecular Sciences*

MDPI

Table S1. Genetic analysis of mutant *lmm24*.

Hybrid Combination	No. of Wild-Type	No. of Mutant	$\chi 2_{0.05} = 3.841$.
ZH8015×*lmm24*	610	206	0.026

Table S2. Primer used in this study for PCR.

Primer	Forward Primer (5′ → 3′)	Reverse Primer (5′ → 3′)	Length of PCR Product
LMM24	ATGCCGCCGAGGCAGTGG	TCAGGGCAGCTTGGCGGA	1344 bp
P-LMM24	TGTGGTCGCTTACTTCGC	TCTCTAAACATTCGCTCTACAAA	5192 bp
GFP-F/R	ATGCCGCCGAGGCAGTGGAG	GGGCAGCTTGGCGGAGGCGC	1341 bp
4th Exons	GCCTCCAACATCCTCCTCG	TACTCGCGATGACGTGGC	735 bp

Table S3. Summary of sequencing data quality.

Sample	Raw Base(bp)	Clean Base(bp)	Effective Rate (%)	Error Rate (%)
pool-WT	6761109300	6754383300	99.91	0.04
pool-M	22194976200	22155026400	99.82	0.04

Table S4. Sequencing depth and coverage statistics.

Sample	Mapped Reads	Total Reads	Mapping Rate (%)	Average Depth (×)
pool-WT	43990838	45031222	97.69	16.1
pool-M	144426427	147700176	97.78	50.98

Table S5. Primer used in this study for gene expression level analysis.

Primer	Forward Primer (5′ → 3′)	Reverse Primer (5′ → 3′)
PR1a	GGCCAATCTCCCTACTGATTAA	GCATAAACACGTAGCATAGCAT
PBZ1	GGTGTGGGAAGCACATACAA	GTCTCCGTCGAGTGTGACTTG
PR1b	TACGCCAGCCAGAGGAGC	GCCGAACCCCAGAAGAGG
PAL1	TTCAACGCCGACACCT	GTAGAGCGGATACGACCTG
AOS2	AAGCTGCTGCAATACGTGTACTGG	CGACGAGCAACAGCCTTCCG
WRKY45	GCCGACGACCAGCACGATCACC	ACGAGCCGACGCCGCCCTC
psaB	TTGGTATTGCTACCGCACAT	CCGGACGTCCATAGAAAGAT
psbA	AAGTTTCTCTGATGGTATG	ATAGCACTGAATAGGGAA
psbB	TCATATTGCTGCGGGTACAT	AGTTGCTGACCCATACCACA
psbC	TACAACCTTGGCAAGAACGA	TACGCCACCCACAGAATTTA
cab2R	GTTCTCCATGTTCGGCTTCT	GACGAAGTTGGTGGCGTAG
rpoA	TCAGGGAATTCACCATGCTA	ATCAAATTGGTCAGGGTGGT
CHLI	AGTAACCTTGGTGCTGTG	AATCCATCAACATTCAACTCTG
CHLD	GGAAAGAGAGGGCATTAG	CAATACGATCAAGTAAGTGTT
SGR	GCAATGTCGCCAAATGACG	GCTCACCACACTCATTCCCTAAAG
Osl2	GCAGACAACAAATCGCCAAAT	TCTCCAGCAACTCTAACCAGCAT
Osl30	AACCTTTTTCTTGGAGATGATACAA	CTTGAACTGTAGGGGCTTGCTT
Osl43	TGTGACAAGTGCTAATAATACATACGA	CCAGACCTTCCAAAGAATCCAAC
Osl85	TCCAGGATGTGATGAGGATTATTC	GCGTGCTGTAGTTCAGTCTGTAAAG
β-actin1	CAGGCCGTCCTCTCTCTGTA	AAGGATAGCATGGGGGAGAG
Ubiquitin	GCTCCGTGGCGGTATCAT	CGGCAGTTGACAGCCCTAG

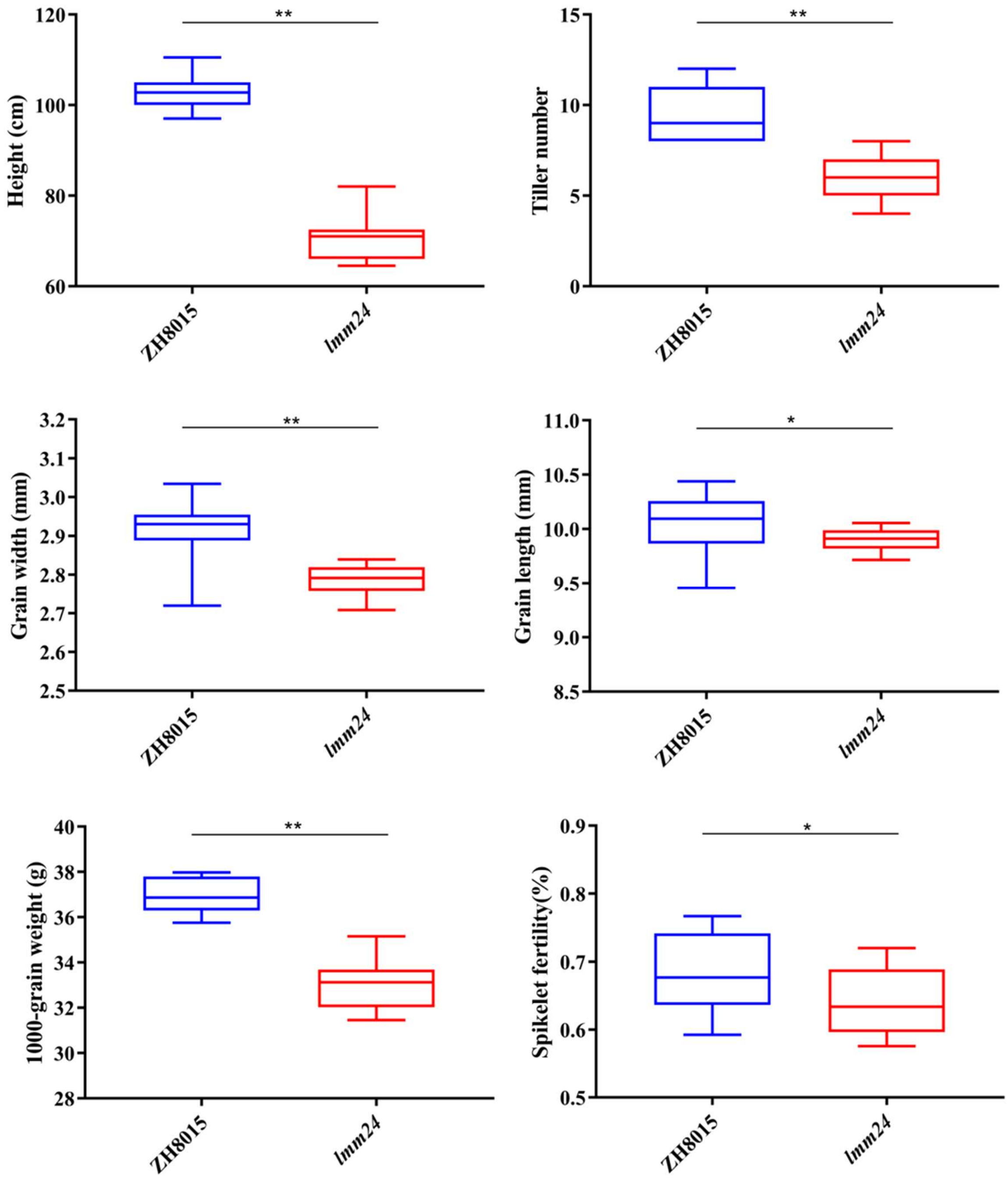

Figure S1. Trait measurements of ZH8015 and *lmm24*. Boxes represent the median values and the first and third quartiles; Whiskers represent the minimum and maximum values. $n = 20$. (Student's *t*-test, *, $p < 0.05$. **, $p < 0.01$).

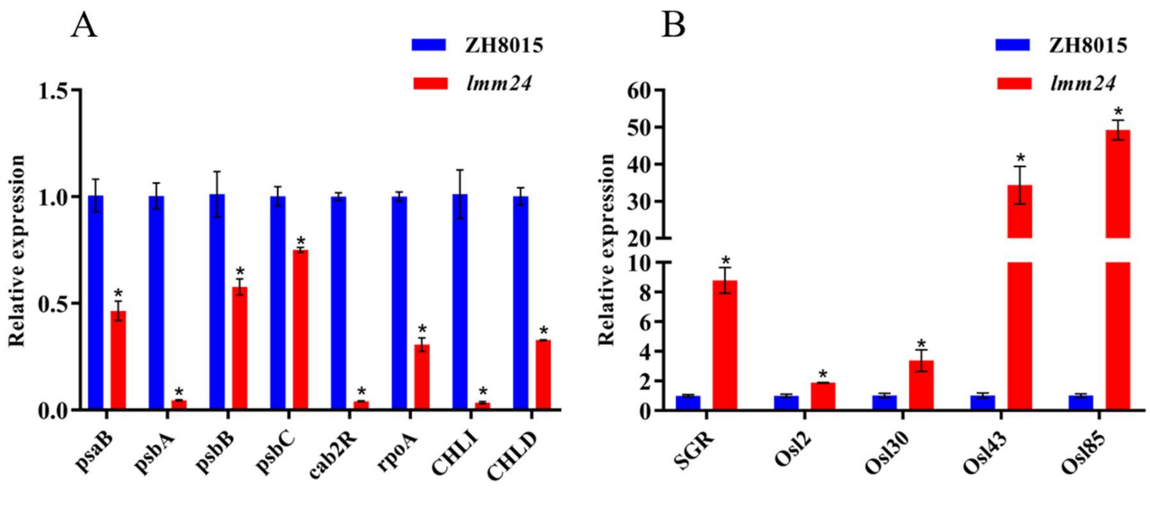

Figure S2. Analysis of expression levels of photosynthesis-related and senescence-related genes, *Ubiquitin* as a reference gene (**A**) Expression levels of photosynthesis-related genes. (**B**) Expression levels of SGR gene and senescence-associated genes. The expression level of each gene in ZH8015 was normalized to 1. Data are means ± SE of three biological replicates. The P value is calculated by the Mann-Whitney U test method. *$p < 0.01$.

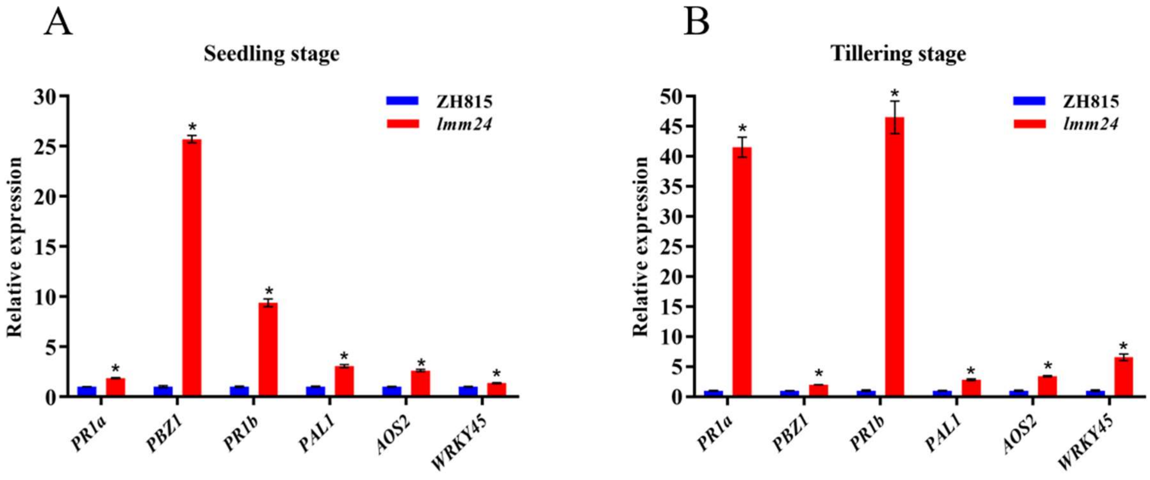

Figure S3. Expression levels of PR genes, *Ubiquitin* as a reference gene. (**A**) Seedling stage (20 dps) (**B**) Tillering stage (50 dps). The expression level of each gene in ZH8015 was normalized to 1. Data are means ± SE of three biological replicates The P value is calculated by the Mann-Whitney U test method. *$p < 0.01$.

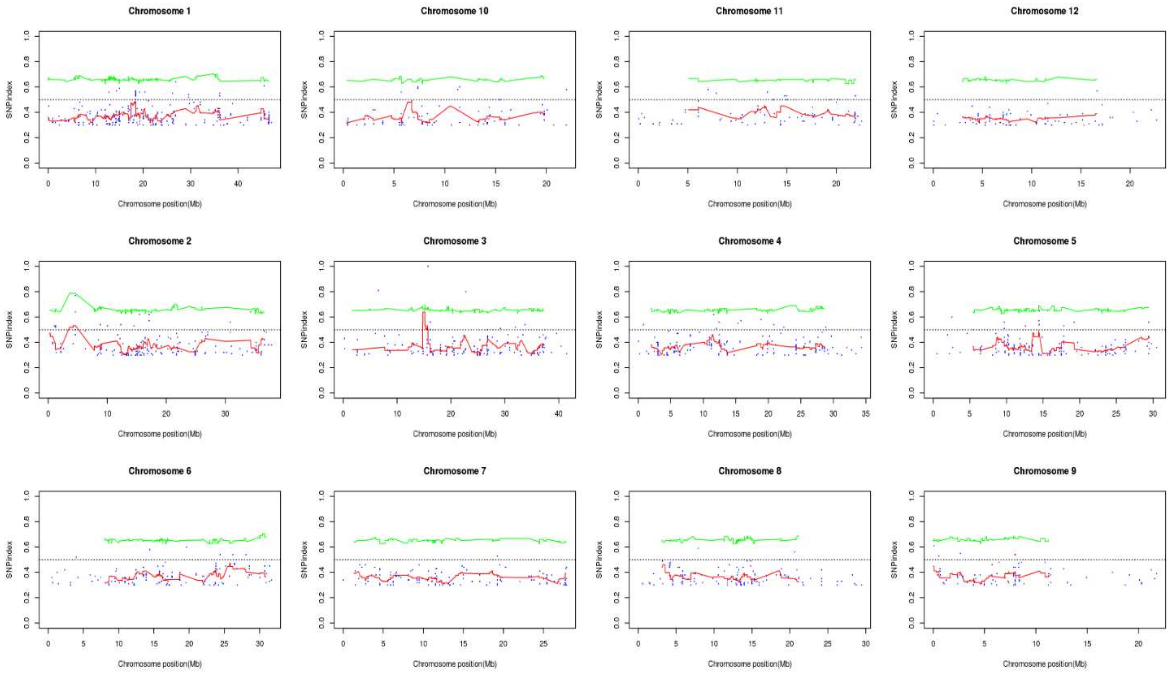

Figure S4. SNP index Manhattan plot of 12 chromosomes in rice. In the Manhattan plot, the X-axis represents the chromosome position, and the Y-axis represents the value of the SNP-index. Each point in the graph represents the position of each candidate site and the value of the SNP-index. The red line indicates the average of the SNP-index of all SNPs in each window. Select 1Mb as the window and 1kb as the step size to calculate the average value of SNP-index in each window.
